# “Once more, with feeling”: no difference in outcomes between patients discharged on oral versus intravenous antibiotics for orthopedic infections in a propensity score matched cohort at a US medical center

**DOI:** 10.1017/ash.2024.57

**Published:** 2024-04-29

**Authors:** Julie Gray, Russell J. Benefield, Chanah K. Gallagher, Heather Cummins, Laura K. Certain

**Affiliations:** 1 Department of Pharmacy, University of Utah Health, Salt Lake City, Utah, USA; 2 Department of Pharmacotherapy, University of Utah College of Pharmacy, Salt Lake City, Utah, USA; 3 University of Utah School of Medicine, Salt Lake City, Utah, USA; 4 Division of Infectious Diseases, University of Utah Health, Salt Lake City, Utah, USA

## Abstract

**Objective:**

To compare outcomes between patients discharged on intravenous (IV) versus oral (PO) antibiotics for the treatment of orthopedic infections, after creation of an IV-to-PO guideline, at a single academic medical center in the United States.

**Methods:**

This was a retrospective, propensity score matched, cohort study of adult patients hospitalized for orthopedic infections from September 30, 2020, to April 30, 2022. Patients discharged on PO antibiotics were matched to patients discharged on IV antibiotics. The primary outcome was one-year treatment failure following discharge. Secondary outcomes were incidence of 60-day treatment failure, adverse drug events (ADE), readmissions, infectious disease clinic “no-show” rates, and emergency department (ED) encounters.

**Results:**

Ninety PO-treated patients were matched to 90 IV-treated patients. Baseline characteristics were similar in the two groups after matching. There was no significant difference in the proportions of patients on PO versus IV antibiotics experiencing treatment failure at one year (26% vs 31%, *P* = .47). There were no significant differences for any secondary outcomes: treatment failure within 60 days (13% vs 14%, *P* = 1.00), ADE (13% vs 11%, *P* = .82), unplanned readmission (17% vs 21%, *P* = .57), or ED encounters (9% vs 18%, *P* = .54). Survival analyses identified no significant differences in time-to-event between PO and IV treatment for any of the outcomes assessed.

**Conclusions:**

There were no appreciable differences in outcomes between patients discharged on PO compared to IV regimens. Antimicrobial stewardship interventions to increase prescribing of PO antibiotics for the treatment of orthopedic infections should be encouraged.

## Introduction

Orthopedic-related infections are a major public health concern because of the high cost of treatment and poor morbidity and mortality outcomes.^1–4^ Historically, the management of orthopedic infections has included a prolonged course of intravenous (IV) antibiotic therapy, typically six weeks.^5,6^ This “standard of care” was based on little data, and continued despite studies suggesting that oral (PO) antibiotic therapy is as effective as intravenous.^7–11^ In 2019, the results from the Oral versus Intravenous Antibiotics for Bone and Joint Infection (OVIVA) trial were published.^12^ This large, randomized controlled trial of over a thousand patients found no difference in the rate of treatment failure between patients given oral compared to IV antibiotics for orthopedic infections.

The advantages of PO over IV antibiotics include both cost and safety. Oral antibiotics by default avoid all catheter-related complications, and are usually less expensive.^13–15^ When institutions have applied the findings of OVIVA to their own practice, in general, they have found reduced costs with similar therapeutic outcomes.^13,16,17^ However, despite the advantages of oral antibiotics and the findings of the OVIVA trial, many clinicians have been reluctant to switch to oral regimens for the treatment of bone and joint infections.^15,18,19^

In autumn 2021, we created an IV-to-PO treatment guideline at our institution, providing guidance to our infectious disease (ID) consult teams about which patients are candidates for oral therapy, and which oral antibiotic regimens are preferred for particular organisms (Supplemental Data). We found that the existence of these guidelines increased the proportion of patients discharged on oral antibiotics for their orthopedic infections.^20^ To evaluate the impact of this change in practice, we conducted a propensity score matched, retrospective cohort study to evaluate the safety and effectiveness of discharging patients on oral antibiotics for orthopedic-related infections compared to intravenous treatment. The goal of the current paper is to add to the literature supporting the use of PO antibiotics for the treatment of orthopedic infections, in the hopes of slowly changing clinical practice through repeated validation of the findings of the OVIVA trial.

## Methods

### Study design and approval

This was a retrospective, observational cohort study with propensity score matching, comparing outcomes between patients treated with IV versus PO antibiotics for bone and joint infections. Patients were identified by searching the University of Utah Health Enterprise Data Warehouse (EDW) for patient encounters associated with surgical procedures performed by the Orthopedic service, or with the ICD-10 diagnosis codes M86XXX (osteomyelitis), T845XXX (infection of prosthetic devices), or M462X (vertebral osteomyelitis), from September 30, 2020, to April 30, 2022. Patients were included if they received at least two consecutive calendar days of IV antibiotics while inpatient, were discharged on an IV or oral antibiotic regimen for a planned total treatment duration ≥14 days for a bone or joint infection, were seen by the ID consult service while inpatient, and had a scheduled ID clinic follow-up visit after discharge. Those inclusion criteria were then confirmed by manual chart review. Patients were excluded if they: were <18 years old; pregnant; discharged to an outside hospital, long-term acute care hospital (LTACH), prison, or hospice; left against medical advice; required chronic renal replacement therapy; or if their planned antibiotic treatment duration was <7 days after discharge. Inclusion was limited to the first eligible encounter for patients with more than one encounter meeting criteria for inclusion during the period of interest. Patients were assigned to their cohort (IV or PO) based on their antibiotic regimen at hospital discharge. Since combination antimicrobial regimens are commonly used for bone and joint infections (eg, adjunctive rifampin), patients treated with more than one agent were included. Patients receiving fully oral combination regimens (eg, levofloxacin and rifampin) were assigned to the PO treatment cohort, while patients receiving partially oral combination regimens (eg, cefazolin and rifampin) were assigned to the IV treatment cohort.

Patient consent was not required for this retrospective chart review study. The study was approved by the University of Utah Health Institutional Review Board under IRB Protocol 00111238.

### Outcomes

The primary outcome was the occurrence of treatment failure within one year of hospital discharge. Treatment failure was defined as a worsening or recurrent infection that required unplanned surgical intervention or antibiotic therapy escalation and was identified by chart review of progress notes. Therapy escalation was defined as extending the duration of therapy beyond the original intended duration, changing to a different antibiotic regimen due to concerns for treatment failure, or reinitiating therapy after a first treatment course, with accompanying progress note documentation from an ID provider of suspected bone or joint infection. Secondary outcomes, assessed within 60 days of hospital discharge, included the occurrence of treatment failure, adverse drug events (ADE), hospital readmission, emergency department (ED) encounters that were not associated with readmission, and ID clinic “no show” visits. ADEs were defined as provider documentation of a suspected medication event combined with discontinuation of the suspected agent. We also collected data on patients who switched from IV to PO regimens prior to the planned end of therapy, and vice versa, along with the reasons for the switch. Patients without a full year of postdischarge follow-up within our medical record were categorized as “lost-to-follow-up.”

### Data collection

Data were collected from the EDW and by chart review by study personnel (JG, RB, HC) using a standard data collection form. Data gathered from the EDW included hospital length of stay, primary hospital service, age, sex assigned at birth, patient-reported race and ethnicity, primary language, discharge disposition, Charlson comorbidity index, primary payor, zip code of primary residence, and hospital readmission or ED encounter within 60 days of discharge.

Data gathered by chart review included discharge antibiotic regimen, antibiotic indication, microbiologic indication, surgical intervention for source control, unhoused status, history of IV drug use, the presence of a chronic infection (defined as an infection lasting ≥6 months, categorized as chronic based on imaging, or a patient previously on suppressive antibiotics), bacteremia, total treatment duration, time to first ID clinic follow-up visit, and the occurrence and time-to-event for the outcomes of treatment failure, ADE, and ID clinic no-show visits. Blinding to treatment assignment was not possible for this retrospective chart review.

### Statistical analysis

Given considerable selection bias in the decision to prescribe oral versus IV treatment for bone and joint infections, a propensity score matching approach was utilized to better estimate the treatment effect between cohorts. Variables expected to be associated with treatment selection and treatment failure, or treatment failure alone, were included in the propensity score model (Table 1). Propensity scores were estimated by logistic regression using the MatchIt package in R.^21^ At first, propensity score matching was performed using a 1:1 nearest neighbor greedy matching algorithm without replacement (ie, each PO-treated patient was matched with the most similar IV-treated patient and patients could not be paired more than once). This led to a suboptimal match result (Supplementary Figure 1), so a caliper term was incorporated into the algorithm to limit the range of acceptable differences in propensity scores in matched patient pairs. An acceptable match quality was found with a caliper distance of 0.25 standard deviations (Supplementary Figure 2), and smaller caliper distances did not appreciably improve the quality of the matched cohorts.

**Table 1.** Baseline characteristics

	Total (*n* = 180)	Intravenous (*n* = 90)	Oral (*n* = 90)
Sex, Male, *n* (%)	111 (62)	47 (52)	64 (71)
Age, mean (SD)^a^	53 (16)	53 (15)	53 (17)
Race, Caucasian, *n* (%)	159 (88)	76 (84)	83 (92)
Ethnicity, Hispanic, *n* (%)	12 (7)	8 (9)	4 (4)
Preferred language other than English, *n* (%)^a^	8 (4)	4 (4)	4 (4)
Payor, *n* (%)^a^			
Private or Workers’ Compensation	86 (48)	41 (46)	45 (50)
Medicare	58 (32)	30 (33)	28 (31)
Medicaid	27 (15)	15 (17)	12 (13)
Other	2 (1)	1 (1)	1 (1)
Uninsured	7 (4)	3 (3)	4 (4)
IV drug use history, *n* (%)^a^	22 (12)	11 (12)	11 (12)
Currently experiencing homelessness, *n* (%)^a^	9 (5)	4 (4)	5 (6)
Charlson comorbidity index, median (IQR)^a^	2 (0–4)	2 (0-5)	2 (0–4)
Hospital length of stay prior to discharge, days, median (IQR)^a^	5.4 (3.1–9.1)	5.9 (3.2–10.5)	4.8 (3.0–8.5)
Duration of follow-up postdischarge, days, median (range)	365 (171–365)	365 (365–365)	365 (78–365)
Distance from primary residence to medical center, miles, median (IQR)	27 (10–130)	26 (11–122)	28 (8–150)
Indication, *n* (%)^a^			
Osteomyelitis	70 (39)	36 (40)	34 (38)
Hardware infection	52 (29)	27 (30)	25 (28)
Prosthetic joint infection	34 (19)	18 (20)	16 (18)
Septic arthritis	26 (14)	11 (12)	15 (17)
Diabetic foot infection	23 (13)	11 (12)	12 (13)
Spondylodiscitis	23 (13)	10 (11)	13 (14)
Bacteremia	15 (8)	5 (6)	10 (11)
Surgical intervention for source control, *n* (%)^a^	151 (84)	76 (84)	75 (83)
Chronic infection, *n* (%)^a^	28 (16)	16 (18)	12 (13)
Site of infection, *n* (%)			
Foot	34 (19)	14 (16)	20 (22)
Knee	30 (17)	15 (17)	15 (17)
Spine	24 (13)	11 (12)	13 (14)
Leg	21 (12)	10 (11)	11 (12)
Ankle	16 (9)	10 (11)	6 (7)
Hip	15 (8)	8 (9)	7 (8)
Shoulder	11 (6)	6 (7)	5 (6)
Other	35 (19)	18 (20)	17 (19)
Causative organism, *n* (%)			
MSSA^a^	46 (26)	21 (23)	25 (28)
Aerobic Gram-negative rod^a^	39 (22)	19 (21)	20 (22)
Enterobacterales	24 (13)	9 (10)	15 (17)
Pseudomonas spp.	12 (7)	6 (7)	6 (7)
Streptococci	29 (16)	13 (14)	16 (18)
Coagulase-negative staphylococci	28 (16)	18 (20)	10 (11)
MRSA^a^	18 (10)	8 (9)	10 (11)
Gram-positive anaerobes	17 (9)	10 (11)	7 (8)
Gram-negative anaerobes	13 (7)	4 (4)	9 (10)
Candida spp.	11 (6)	3 (3)	8 (9)
Enterococcus spp.	12 (7)	9 (10)	3 (3)
Cutibacterium spp.	6 (3)	2 (2)	4 (4)
Culture negative^a^	35 (19)	18 (20)	17 (19)
Multi-drug regimen, *n* (%)	86 (48)	45 (50)	41 (46)
Total duration of therapy, days, median (IQR)	39 (31–76)	39 (34–77)	39 (29–76)
Post-IV-to-PO guideline, *n* (%)^a^	66 (37)	32 (36)	34 (38)
Discharge Disposition, *n* (%)			
Home	132 (73)	63 (70)	69 (77)
Facility	48 (27)	27 (30)	21 (23)
Hospital service^a^			
Orthopedics	85 (47)	43 (48)	42 (47)
Internal medicine	61 (34)	29 (32)	32 (36)
Plastic surgery	16 (9)	8 (9)	8 (9)
Other	18 (10)	10 (11)	8 (9)

Abbreviations: IV, intravenous; SD, standard deviation; IQR, interquartile range; MRSA, methicillin-resistant *Staphylococcus aureus*; MSSA, methicillin-susceptible *Staphylococcus aureus.*

a Variable included in propensity score matching.

Total counts and percentages, median and interquartile ranges, and means and standard deviations were used to summarize data as appropriate. Inferential tests of significance were avoided for descriptive data tables.^22^ Patients without a documented substance abuse history were considered to have no history of IV drug use; no other adjustments were necessary to account for missing data as all other data were gathered completely. No formal power calculation was determined *a priori*; a convenience sample of all eligible patients was included. Differences in proportions were determined for each outcome and Wald-based 95% confidence intervals that considered the IV and PO cohorts as paired were determined using the Misty package in R (Yanagida T, “Package ‘misty’” Version 0.5.3. 2023-09-17). Differences in outcomes between IV- and PO-treated patients were considered statistically significant if the 95% confidence intervals did not contain zero. *P*-values for univariate comparisons were determined using McNemar’s test.

Survival functions for time-to-treatment failure by IV versus PO treatment were estimated using the Kaplan-Meier method and assessed for significance using the log-rank test. Time zero was defined as the date of hospital discharge, and patients were assessed from the time of discharge until they experienced treatment failure or up to one year following discharge. Secondary outcomes were assessed up to 60 days after discharge. Patients were censored at the date of last observation in our health system if less than one year postdischarge.

Statistical analyses were completed using JMP Pro version 16.1.0 (SAS Institute, Cary, NC) and R Statistical Software version 4.2.2 (R Core Team 2022).

## Results

Six hundred twelve unique inpatient encounters were screened and 358 met criteria for inclusion. Reasons for exclusion included: antibiotic indication other than acute treatment of bone or joint infection (*n* = 133); discharge to outside hospital, LTACH, prison, or hospice (*n* = 48); no planned ID clinic follow-up or follow-up with outside provider (*n* = 24); antibiotic duration less than two weeks total or less than seven days postdischarge (*n* = 24); chronic renal replacement therapy (*n* = 14); left against medical advice (*n* = 10); antibiotics initiated while outpatient (*n* = 1). One hundred seventeen PO- and 241 IV-treated patients were eligible for inclusion. After propensity score matching, 90 patients from each cohort remained for further analysis. Baseline characteristics between the two treatment groups were similar after matching, although a greater proportion of patients treated with oral regimens were male (Table 1). Methicillin-sensitive *Staphylococcus aureus* (MSSA) was the most common organism included in the matched cohort; approximately 20% of patients were treated for culture-negative infections. Roughly half of the cohort were treated with combination regimens, regardless of IV or PO treatment. The median (IQR) time to first ID clinic follow-up visit was 16 days (11–25 days), and was similar in the IV (17 days, IQR 11–27) and oral (15 days, IQR 12–24) cohorts.

Fifty-seven patients were censored prior to one year of observation after discharge: 20 in the IV group and 37 in the oral group. A greater proportion of patients treated with oral regimens were lost to follow-up prior to one year compared to patients treated with IV regimens (23% vs 8%, *P* = .008). Other reasons for censoring were similar between cohorts and included no need for further follow-up (*n* = 20, 9 IV and 11 PO) and patient death (*n* = 9, 4 IV and 5 PO). The median follow-up time in both cohorts was 365 days.

Cephalosporins (51%) and vancomycin (41%) were the most common IV agents included in the matched cohort. The most common PO agents were fluoroquinolones (40%), followed by amoxicillin or amoxicillin-clavulanate (29%), trimethoprim-sulfamethoxazole (24%), and doxycycline (19%). (Supplementary Table 1)

Fifty-one patients (28%) failed treatment within one year of hospital discharge (Table 2). There was no significant difference observed in the proportion of patients experiencing treatment failure at one year in the PO (26%) compared to IV (31%) treatment groups (difference –5.6%, 95% CI: –17.6% to 6.5%). Since a greater number of patients were lost to follow-up prior to one year of observation in the PO compared to IV cohort, two *post hoc* sensitivity analyses were completed. When all patients lost to follow-up were considered treatment failures there remained no significant difference between treatment groups [37% IV versus 41% PO; difference 4.4% (95% CI: –9.0% to 17.8%)]. When all patients treated with oral regimens that were lost to follow-up were counted as treatment failures and all IV patients lost to follow-up were considered successes, the apparent difference in one-year failure was 31% in the IV group compared to 41% in the oral group [difference 10.0% (95% CI: –3.1% to 23.1%)].

**Table 2.** Univariate comparisons

	Total (*n* = 180)	Intravenous (*n* = 90)	Oral (*n* = 90)	*P*-value	Difference in Proportions (95% Confidence Interval)^a^
Treatment failure within 365 days, *n* (%)	51 (28)	28 (31)	23 (26)	0.47	−5.6% (−17.6% to 6.5%)
Treatment failure within 60 days, *n* (%)	25 (14)	13 (14)	12 (13)	1.00	−1.1% (−11.1% to 8.9%)
Adverse drug event within 60 days, *n* (%)	22 (12)	10 (11)	12 (13)	0.82	2.2% (−7.5% to 12.0%)
Unplanned readmission within 60 days, *n* (%)	34 (19)	19 (21)	15 (17)	0.57	−4.4% (−15.9% to 7.0%)
ED encounter within 60 days, *n* (%)	24 (13)	16 (18)	8 (9)	0.15	−8.9% (−19.4% to 1.6%)
No show follow-up visit, *n* (%)	28 (16)	12 (13)	16 (18)	0.54	4.4% (−6.2% to 15.1%)

a Differences reported are for oral treatment compared to intravenous.

**Figure 1.**
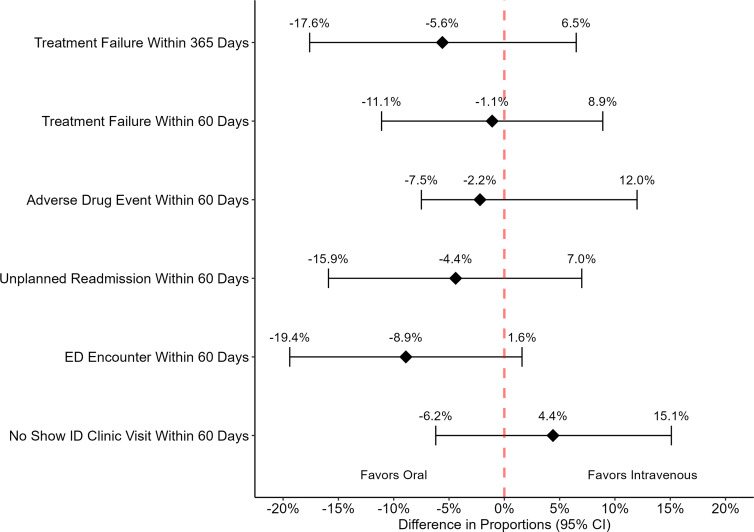
Forest plot of differences in outcomes.

**Figure 2.**
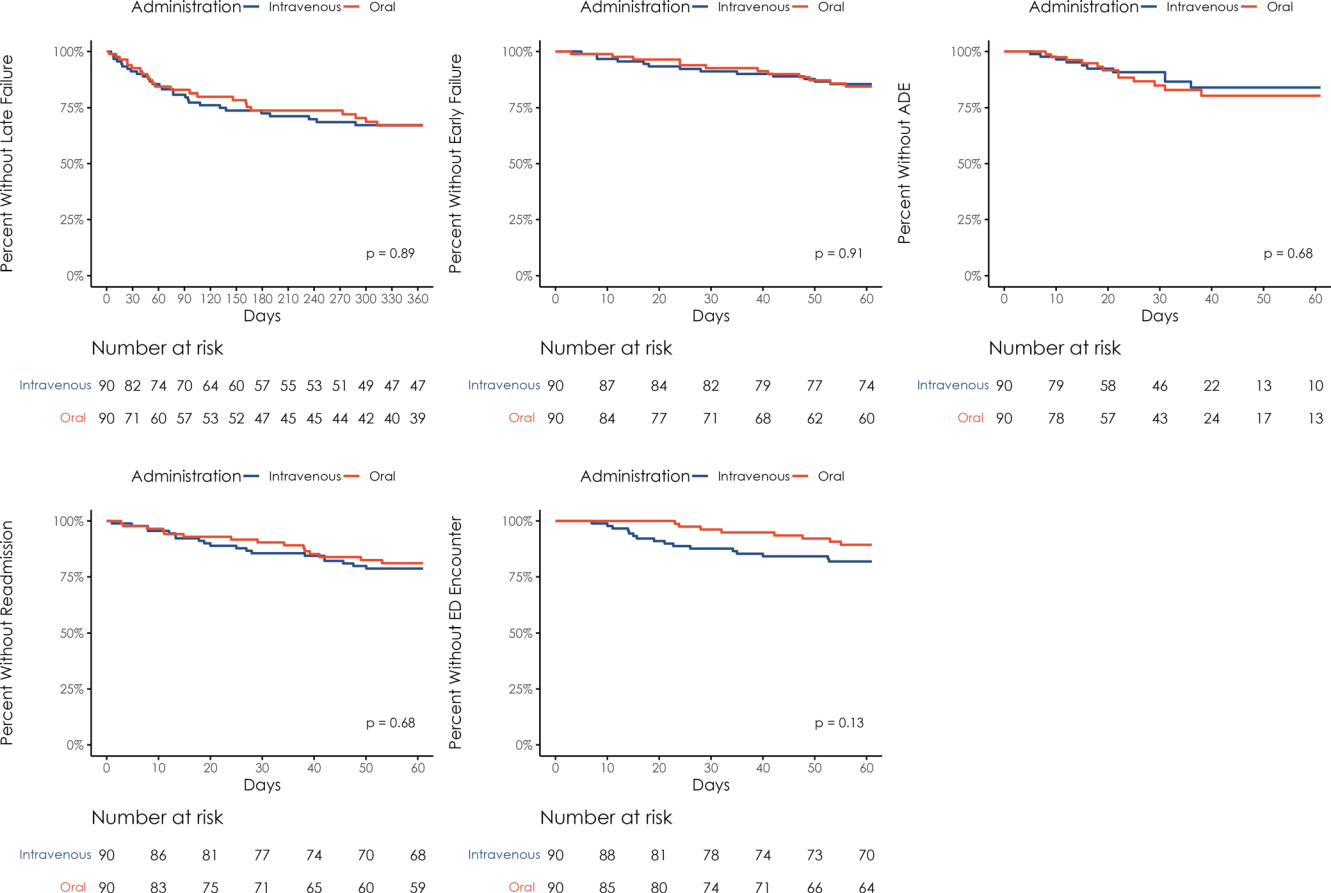
Kaplan-Meier survival curves of time-to-event for outcomes.

There were also no significant differences in the proportions of patients experiencing any of the secondary outcomes that were assessed (Figure 1). Although not statistically significant, twice as many patients in the IV (16) compared to PO (8) cohort experienced an ED encounter within 60 days that was not associated with readmission. Closer *post hoc* inspection of these encounters found that 15 encounters were related to infection or antibiotics (11 due to concern for treatment failure, 2 vascular access complications, 1 patient refusal of IV OPAT), and infection/antibiotic-related ED encounters were more prevalent in the IV treatment cohort (12 vs 3 patients). Survival analyses found no difference in time-to-event for any of the outcomes assessed between the PO and IV treatment cohorts (Figure 2).

Twenty-eight patients initially discharged on IV regimens were transitioned to oral regimens prior to their intended discontinuation date, most commonly due to de-escalating therapy at an ID clinic follow-up visit (*n* = 17). Other reasons for a change from IV to PO therapy included: extending the planned treatment course with an oral regimen (3), transitioning from induction therapy to consolidation or chronic suppression with an oral regimen (3), an adverse event to the IV agent (2), loss of central vascular access (1), patient refusal of continued IV treatment (1), and tenuous housing status (1). Nine patients discharged on oral regimens were changed to IV regimens after discharge. Eight of these patients were switched to IV regimens to broaden antimicrobial spectrum in response to new or worsening infections, and one patient switched to IV in response to bone marrow suppression with oral linezolid. Four patients switched from PO to IV antibiotics due to concern for worsening infection required further surgery.

## Discussion

Using a propensity score matched, retrospective cohort study design, we found no difference in outcomes for patients treated with PO versus IV antibiotics for orthopedic infections. One-year treatment failure was similar between groups, and there was likewise no significant difference between the two groups for 60-day treatment failure, readmissions, ED visits, adverse events, or ID clinic “no-show” appointments. These results add to the growing body of literature demonstrating the safety and efficacy of using oral antibiotics for the treatment of serious infections and refute the concern that patients discharged on oral antibiotics are less likely to keep their appointments or take their medications.

In recent years there has been a push to reconsider the dogma surrounding treatment of orthopedic infections, and increasing attention on the lack of data to support our ingrained preference for IV antibiotics.^6^ The OVIVA trial provided clear data that PO antibiotics are as effective as IV antibiotics for the treatment of orthopedic infections, leading many centers (including ours) to start using oral antibiotic regimens more frequently. Several institutions have previously published their experience using oral antibiotics, either to treat orthopedic infections in general^13,14,23^ or in specific situations.^24–26^ Similar to the current study, many of these institutions made a concerted effort to consider oral antibiotics for the treatment of orthopedic infections and saw a subsequent increase in the proportion of patients discharged on oral antibiotics. Also similar to the current study, no studies have found any increased risk of treatment failure when using PO versus IV antibiotics. Our study adds to this literature and has the strength of using a propensity score matched cohort to control for selection bias in choosing oral versus IV treatment.

While our results are overall consistent with studies at other institutions, there are notable limitations to consider when interpreting our analysis. Retrospective cohorts are inherently limited by selection bias. We attempted to decrease this bias through propensity score matching, but one consequence of this approach is that these results may be less generalizable. Propensity score matching also resulted in a smaller sample size, which increases the potential for a type II error. For example, the finding that patients discharged on IV antibiotics had twice the number of ED visits compared to patients discharged on PO antibiotics, while not statistically significant, may reflect a true difference given the known complications associated with central lines. In addition, despite propensity matching, there is likely some residual confounding due to selection bias. A clinician’s perception of illness severity and risk of infection relapse may influence their choice of IV versus PO antibiotics. While we did our best to capture severity of illness with factors such as hospital length of stay and Charlson comorbidity index, we did not include specific measures of severity of illness *per se* in our matching. There were more patients without a full year of follow-up in the PO group. This difference may reflect less illness severity in this group, though may also be due to physicians choosing PO antibiotics for patients that they think, for whatever reason, will have difficulty staying engaged with the healthcare system.

Another limitation is that our results are heavily dependent on accurate provider documentation of both primary and secondary outcomes. Also, patients were not limited to receiving either IV or PO antibiotics for the entirety of their treatment duration. Some of those discharged on IV therapy were switched to PO treatment after discharge, and vice versa; our choice to assign outcomes to the discharge antibiotic regimen could have led to misattribution of treatment failure or adverse events. However, our results are in agreement with the OVIVA trial as well as other single-center cohorts. Despite some limitations, our data indicate discharging patients with orthopedic infections on PO therapy results in similar outcomes to providing patients with IV antibiotics at discharge. We were not able to analyze which PO regimens are optimal due to heterogeneity (Supplemental Tables 2 and 3) but we hope that will be the focus of further study.

In conclusion, patients discharged on oral antibiotics had similar outcomes as those who were discharged on IV therapy. The results of this retrospective, propensity score matched, cohort study were similar to other published studies on this topic. Antimicrobial stewardship interventions to increase prescribing of PO antibiotics for the treatment of orthopedic infections, such as the implementation of institutional guidelines, should be encouraged.

## Supporting information

Gray et al. supplementary material 1 Gray et al. supplementary material

Gray et al. supplementary material 2 Gray et al. supplementary material

Gray et al. supplementary material 3 Gray et al. supplementary material

## Data Availability

Deidentified data is available from the authors upon request.

## Supplementary material

To view supplementary material for this article, please visit https://doi.org/10.1017/ash.2024.57
