# Supplementary material for: “Once more, with feeling”: no difference in outcomes between patients discharged on oral versus intravenous antibiotics for orthopedic infections in a propensity score matched cohort at a US medical center
Source: Antimicrob Steward Healthc Epidemiol. 2024 Apr 29;4(1):e61. doi: 10.1017/ash.2024.57 (PMC11062794; doi:10.1017/ash.2024.57)
Supplement: Gray et al. supplementary material 1 — Gray et al. supplementary material [file S2732494X24000573sup001.docx]

**Supplementary Table 1: Discharge antibiotics**

|  | **Total**  **(n = 180)** | **Intravenous**  **(n = 90)** | **Oral**  **(n=90)** |
| --- | --- | --- | --- |
| Beta-lactam | 96 (53) | 65 (72) | 31 (34) |
| Cephalosporin | 51 (28) | 46 (51) | 5 (6) |
| Penicillin | 36 (20) | 10 (11) | 26 (29) |
| Carbapenem | 9 (5) | 9 (10) | - |
| Fluoroquinolone | 45 (25) | 9 (10) | 36 (40) |
| Vancomycin | 38 (21) | 38 (42) | - |
| Rifampin | 23 (13) | 13 (14) | 10 (11) |
| Trimethoprim-sulfamethoxazole | 23 (13) | 1 (1) | 22 (24) |
| Tetracycline | 19 (11) | 2 (22) | 17 (19) |
| Fluconazole | 11 (6) | 4 (4) | 7 (8) |
| Metronidazole | 9 (5) | 5 (6) | 4 (4) |
| Linezolid | 5 (3) | 0 (0) | 5 (6) |
| Daptomycin | 3 (2) | 3 (3) | - |
