# Supplementary material for: “Once more, with feeling”: no difference in outcomes between patients discharged on oral versus intravenous antibiotics for orthopedic infections in a propensity score matched cohort at a US medical center
Source: Antimicrob Steward Healthc Epidemiol. 2024 Apr 29;4(1):e61. doi: 10.1017/ash.2024.57 (PMC11062794; doi:10.1017/ash.2024.57)
Supplement: Gray et al. supplementary material 2 — Gray et al. supplementary material [file S2732494X24000573sup002.docx]

**Supplementary Table 2: Outcomes Associated with Oral Antibiotic Regimens**

| **Oral Regimen** | **Number of Patients** | **Treatment Success at 365 Days** | **Adverse Drug Event Within 60 Days** | **Unplanned Readmission Within 60 Days** | **ED Encounter Within 60 Days** | **No Show Follow-up Visit** |
| --- | --- | --- | --- | --- | --- | --- |
| Amoxicillin | 2 | 2 | 0 | 0 | 0 | 0 |
| Amoxicillin and  Trimethoprim-Sulfamethoxazole | 1 | 1 | 0 | 0 | 0 | 0 |
| Amoxicillin-clavulanate | 8 | 4 | 0 | 2 | 2 | 2 |
| Amoxicillin-clavulanate and Ciprofloxacin | 2 | 2 | 0 | 1 | 0 | 0 |
| Amoxicillin-clavulanate and Doxycycline | 8 | 7 | 0 | 0 | 0 | 0 |
| Amoxicillin-clavulanate, Doxycycline, and Posaconazole | 1 | 1 | 0 | 0 | 0 | 0 |
| Amoxicillin-clavulanate and Fluconazole | 2 | 1 | 0 | 1 | 0 | 0 |
| Amoxicillin-clavulanate and Levofloxacin | 1 | 0 | 1 | 1 | 1 | 0 |
| Amoxicillin-clavulanate and Trimethoprim-Sulfamethoxazole | 1 | 1 | 0 | 1 | 1 | 1 |
| Cefixime | 1 | 1 | 0 | 0 | 0 | 1 |
| Cefuroxime | 2 | 2 | 1 | 0 | 0 | 0 |
| Cefuroxime and Doxycycline | 1 | 1 | 1 | 0 | 0 | 0 |
| Cephalexin | 1 | 1 | 0 | 0 | 0 | 0 |
| Ciprofloxacin | 3 | 3 | 0 | 1 | 0 | 1 |
| Clindamycin and Levofloxacin | 1 | 1 | 0 | 0 | 0 | 1 |
| Doxycycline | 1 | 1 | 0 | 0 | 0 | 0 |
| Doxycycline and Levofloxacin | 5 | 2 | 1 | 2 | 0 | 1 |
| Doxycycline and Rifampin | 1 | 1 | 0 | 0 | 0 | 0 |
| Fluconazole | 1 | 0 | 0 | 0 | 0 | 0 |
| Levofloxacin | 10 | 6 | 0 | 2 | 2 | 1 |
| Levofloxacin and Fluconazole | 3 | 3 | 1 | 1 | 0 | 0 |
| Levofloxacin and Metronidazole | 2 | 2 | 0 | 0 | 0 | 1 |
| Levofloxacin and Rifampin | 6 | 5 | 2 | 0 | 0 | 1 |
| Levofloxacin and  Trimethoprim-Sulfamethoxazole | 2 | 2 | 0 | 0 | 0 | 0 |
| Levofloxacin and Voriconazole | 1 | 1 | 0 | 0 | 0 | 1 |
| Linezolid | 3 | 3 | 0 | 0 | 0 | 1 |
| Linezolid and Fluconazole | 1 | 0 | 0 | 0 | 1 | 0 |
| Linezolid and Rifampin | 1 | 0 | 1 | 1 | 0 | 0 |
| Trimethoprim-Sulfamethoxazole | 14 | 10 | 3 | 2 | 1 | 4 |
| Trimethoprim-Sulfamethoxazole and Metronidazole | 2 | 1 | 0 | 0 | 0 | 0 |
| Trimethoprim-Sulfamethoxazole and Rifampin | 2 | 2 | 1 | 0 | 0 | 0 |
| **Total** | **90** | **67** | **12** | **15** | **8** | **16** |

**Supplementary Table 3: Outcomes Associated with Intravenous Antibiotic Regimens**

| **Intravenous Regimen** | **Number of Patients** | **Treatment Success** | **Adverse Event** | **Readmission** | **ED Encounter** | **No Show** |
| --- | --- | --- | --- | --- | --- | --- |
| Cefazolin | 17 | 11 | 2 | 4 | 2 | 2 |
| Cefazolin and Doxycycline (PO) | 1 | 1 | 0 | 0 | 0 | 0 |
| Cefazolin and Levofloxacin (PO) | 1 | 0 | 0 | 0 | 0 | 0 |
| Cefazolin and Rifampin (PO) | 7 | 5 | 1 | 3 | 1 | 0 |
| Cefepime | 4 | 4 | 0 | 0 | 0 | 0 |
| Cefepime and Vancomycin | 1 | 1 | 0 | 1 | 0 | 0 |
| Cefepime, Daptomycin, and Metronidazole (PO) | 1 | 1 | 0 | 0 | 0 | 1 |
| Cefepime and  Trimethoprim-Sulfamethoxazole (PO) | 1 | 1 | 0 | 0 | 0 | 0 |
| Ceftriaxone | 2 | 1 | 0 | 0 | 0 | 0 |
| Ceftriaxone and Metronidazole (PO) | 1 | 1 | 0 | 1 | 0 | 0 |
| Ceftriaxone and Vancomycin | 7 | 7 | 0 | 1 | 0 | 1 |
| Ceftriaxone, Vancomycin, and Fluconazole (PO) | 1 | 1 | 0 | 0 | 0 | 1 |
| Daptomycin | 1 | 1 | 0 | 0 | 0 | 0 |
| Daptomycin, Piperacillin-tazobactam, and Fluconazole (PO) | 1 | 1 | 1 | 0 | 0 | 0 |
| Ertapenem | 2 | 1 | 0 | 1 | 2 | 0 |
| Ertapenem and Vancomycin | 1 | 1 | 1 | 0 | 0 | 0 |
| Ertapenem and Fluconazole (PO) | 1 | 1 | 0 | 0 | 1 | 0 |
| Meropenem | 3 | 2 | 0 | 0 | 1 | 0 |
| Meropenem and Vancomycin | 2 | 1 | 0 | 1 | 1 | 0 |
| Penicillin | 3 | 1 | 0 | 1 | 1 | 1 |
| Piperacillin-tazobactam | 4 | 2 | 0 | 0 | 1 | 1 |
| Piperacillin-tazobactam and Doxycycline (PO) | 1 | 1 | 0 | 0 | 1 | 0 |
| Piperacillin-tazobactam and  Rifampin (PO) | 1 | 1 | 1 | 0 | 0 | 0 |
| Vancomycin | 8 | 5 | 0 | 1 | 3 | 2 |
| Vancomycin and Fluconazole (PO) | 1 | 0 | 0 | 1 | 0 | 1 |
| Vancomycin and Levofloxacin (PO) | 7 | 6 | 2 | 0 | 2 | 0 |
| Vancomycin and Metronidazole (PO) | 1 | 1 | 0 | 0 | 0 | 0 |
| Vancomycin and Rifampin (PO) | 5 | 1 | 1 | 2 | 0 | 1 |
| Vancomycin and Voriconazole (PO) | 1 | 0 | 0 | 0 | 0 | 0 |
| Vancomycin, Cefepime, and Metronidazole (PO) | 1 | 1 | 0 | 1 | 0 | 1 |
| Vancomycin, Levofloxacin (PO), and Metronidazole (PO) | 1 | 0 | 1 | 1 | 0 | 0 |
| Vancomycin and  Piperacillin-tazobactam | 1 | 1 | 0 | 0 | 0 | 0 |
| **Total** | **90** | **62** | **10** | **19** | **16** | **12** |
