# Supplementary material for: “Once more, with feeling”: no difference in outcomes between patients discharged on oral versus intravenous antibiotics for orthopedic infections in a propensity score matched cohort at a US medical center
Source: Antimicrob Steward Healthc Epidemiol. 2024 Apr 29;4(1):e61. doi: 10.1017/ash.2024.57 (PMC11062794; doi:10.1017/ash.2024.57)
Supplement: Gray et al. supplementary material 3 — Gray et al. supplementary material [file S2732494X24000573sup003.docx]

STROBE Statement—checklist of items that should be included in reports of observational studies

|  | Item No. | Recommendation | Page  No. | Relevant text from manuscript |
| --- | --- | --- | --- | --- |
| **Title and abstract** | 1 | (*a*) Indicate the study’s design with a commonly used term in the title or the abstract | 1 | “propensity score matched cohort” |
|  |  | (*b*) Provide in the abstract an informative and balanced summary of what was done and what was found | 2 | Abstract |
| Introduction | | | |  |
| Background/rationale | 2 | Explain the scientific background and rationale for the investigation being reported | 3 | Introduction |
| Objectives | 3 | State specific objectives, including any prespecified hypotheses | 3 | Last sentence of the introduction. |
| Methods | | | |  |
| Study design | 4 | Present key elements of study design early in the paper | 3-4 | “Study Design and Approval” section of Methods |
| Setting | 5 | Describe the setting, locations, and relevant dates, including periods of recruitment, exposure, follow-up, and data collection | 3-4 | “Study Design and Approval” section of Methods |
| Participants | 6 | (*a*) *Cohort study*—Give the eligibility criteria, and the sources and methods of selection of participants. Describe methods of follow-up  *Case-control study*—Give the eligibility criteria, and the sources and methods of case ascertainment and control selection. Give the rationale for the choice of cases and controls  *Cross-sectional study*—Give the eligibility criteria, and the sources and methods of selection of participants | 3-4 | “Study Design and Approval” section of Methods |
|  |  | (*b*) *Cohort study*—For matched studies, give matching criteria and number of exposed and unexposed  *Case-control study*—For matched studies, give matching criteria and the number of controls per case | 5 | First paragraph of “Statistical Analysis” section of Methods |
| Variables | 7 | Clearly define all outcomes, exposures, predictors, potential confounders, and effect modifiers. Give diagnostic criteria, if applicable | 4 | Outcomes defined in “Outcomes” section of Methods and the primary predictor assessed was IV versus oral treatment. |
| Data sources/ measurement | 8* | For each variable of interest, give sources of data and details of methods of assessment (measurement). Describe comparability of assessment methods if there is more than one group | 5 | “Data Collection” section of Methods |
| Bias | 9 | Describe any efforts to address potential sources of bias | 5 | Propensity score matching described in “Statistical Analysis” section of Methods |
| Study size | 10 | Explain how the study size was arrived at | 5 | Convenience sample described in second paragraph of “Statistical Analysis” description in Methods |

| Quantitative variables | 11 | Explain how quantitative variables were handled in the analyses. If applicable, describe which groupings were chosen and why | 5-6 | “Statistical Analysis” section of Methods |
| --- | --- | --- | --- | --- |
| Statistical methods | 12 | (*a*) Describe all statistical methods, including those used to control for confounding | 5-6 | “Statistical Analysis” section of Methods |
|  |  | (*b*) Describe any methods used to examine subgroups and interactions | N/A |  |
|  |  | (*c*) Explain how missing data were addressed | 5 | Second paragraph of “Statistical Analysis” section of Methods |
|  |  | (*d*) *Cohort study*—If applicable, explain how loss to follow-up was addressed  *Case-control study*—If applicable, explain how matching of cases and controls was addressed  *Cross-sectional study*—If applicable, describe analytical methods taking account of sampling strategy | 5 | Fourth paragraph of “Statistical Analysis” section of Methods |
|  |  | (*e*) Describe any sensitivity analyses | 7 | “two post hoc sensitivity analyses were completed” |
| Results | | | | |
| Participants | 13* | (a) Report numbers of individuals at each stage of study—eg numbers potentially eligible, examined for eligibility, confirmed eligible, included in the study, completing follow-up, and analysed | 6 | First paragraph of Results |
|  |  | (b) Give reasons for non-participation at each stage | 6-7 | First paragraph of Results |
|  |  | (c) Consider use of a flow diagram | N/A | Reported in text due to figure limitations |
| Descriptive data | 14* | (a) Give characteristics of study participants (eg demographic, clinical, social) and information on exposures and potential confounders | 6-7 | First paragraph of Results as well as Table 1 |
|  |  | (b) Indicate number of participants with missing data for each variable of interest | N/A |  |
|  |  | (c) *Cohort study*—Summarise follow-up time (eg, average and total amount) | 7 | Second paragraph of results |
| Outcome data | 15* | *Cohort study*—Report numbers of outcome events or summary measures over time | 7-8 | Final paragraph of Results |
|  |  | *Case-control study—*Report numbers in each exposure category, or summary measures of exposure |  |  |
|  |  | *Cross-sectional study—*Report numbers of outcome events or summary measures |  |  |
| Main results | 16 | (*a*) Give unadjusted estimates and, if applicable, confounder-adjusted estimates and their precision (eg, 95% confidence interval). Make clear which confounders were adjusted for and why they were included | 8 | Final paragraph of Results along with Figures 1 and 2 |
|  |  | (*b*) Report category boundaries when continuous variables were categorized | N/A |  |
|  |  | (*c*) If relevant, consider translating estimates of relative risk into absolute risk for a meaningful time period | N/A |  |

| Other analyses | 17 | Report other analyses done—eg analyses of subgroups and interactions, and sensitivity analyses | 7 | “two post hoc sensitivity analyses were completed” |
| --- | --- | --- | --- | --- |
| Discussion | | | | |
| Key results | 18 | Summarise key results with reference to study objectives | 8 | First paragraph of Discussion |
| Limitations | 19 | Discuss limitations of the study, taking into account sources of potential bias or imprecision. Discuss both direction and magnitude of any potential bias | 9 | Last paragraph of Discussion |
| Interpretation | 20 | Give a cautious overall interpretation of results considering objectives, limitations, multiplicity of analyses, results from similar studies, and other relevant evidence | 8-9 | Second paragraph of Discussion |
| Generalisability | 21 | Discuss the generalisability (external validity) of the study results | 9 | Last paragraph of Discussion |
| Other information | |  | | |
| Funding | 22 | Give the source of funding and the role of the funders for the present study and, if applicable, for the original study on which the present article is based | N/A | Unfunded work |

*Give information separately for cases and controls in case-control studies and, if applicable, for exposed and unexposed groups in cohort and cross-sectional studies.

**Note:** An Explanation and Elaboration article discusses each checklist item and gives methodological background and published examples of transparent reporting. The STROBE checklist is best used in conjunction with this article (freely available on the Web sites of PLoS Medicine at http://www.plosmedicine.org/, Annals of Internal Medicine at http://www.annals.org/, and Epidemiology at http://www.epidem.com/). Information on the STROBE Initiative is available at www.strobe-statement.org.
